# Robustness and Specificity in Signal Transduction via Physiologic Protein Interaction Networks

**DOI:** 10.4172/2161-1459.S3-001

**Published:** 2012-12-21

**Authors:** Adam G. Schrum, Diana Gil

**Affiliations:** Department of Immunology, Mayo Clinic College of Medicine, Rochester, Minnesota, USA

## Abstract

The collective Protein:Protein Interactions (PPI) of a cell are thought to represent a system with emergent network properties that integrate signals from a multiplicity of inputs into coordinated responses. It is hypothesized that the PPI network supplies both specificity for many distinct signals that utilize common intermediate pathways, and also robustness by allowing specific signals to be communicated by alternate routes. Progress with genetic networks points to these concepts, but the extent to which PPI networks possess these properties has not been empirically tested, due to lack of quantitative data needed for such assessments. Here, a hypothetical physiologic PPI network is used to illustrate how signaling robustness and specificity could be manifest under conditions of (i) deletion mutation, or (ii) changes in signaling due to variation in environmental conditions or stimuli. It is proposed that advances in technology enabling empirical analysis of PPI network principles will have the potential to significantly impact basic understanding of signaling mechanisms, and contribute to the generation of novel applications in drug screening and pharmacology.

## Signal Specificity Through Protein:Protein Interaction (PPI) Networks

**Figure 1:**
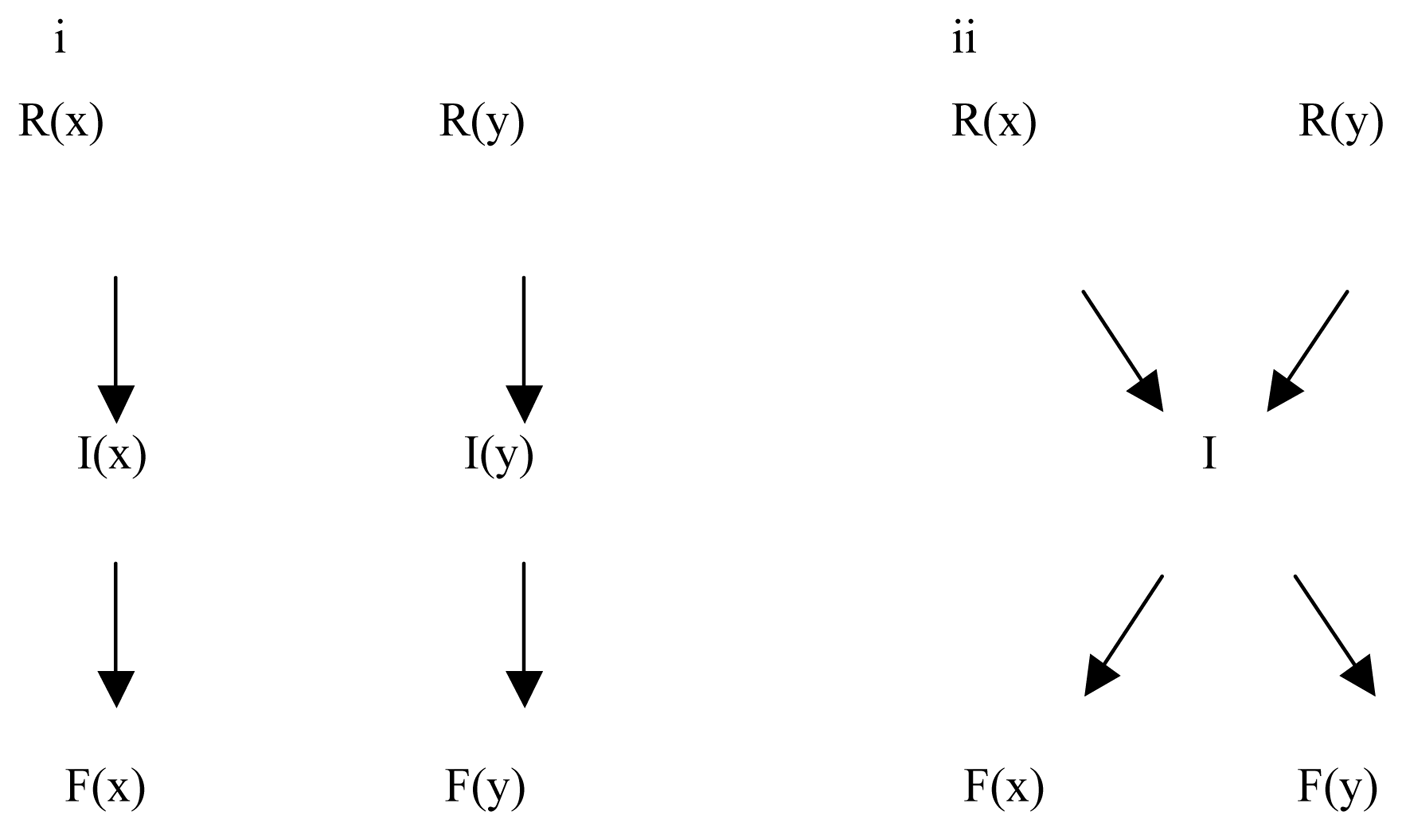
Two distinct mechanisms to achieve signal specificity **(i)** Two unique cellular signals (x,y) are transmitted via their own unique receptors (R) and intermediates (I), to effect distinct cellular functions (F). **(ii)** Signals x and y are transmitted through unique receptors, but they use common intermediates to effect unique functions.

A basic aspect of cell physiology involves the ability to generate specific responses to specific stimuli via signal transduction. The principle that receptor:ligand interactions recognize and initiate these specific signals is well established. However, it is much less clear how the immediate next step in the process, participation of intracellular intermediates, communicates the specificity of the original interaction to produce the ‘correct’ cellular response. In principle, this could be achieved via receptor-customized intracellular intermediates that would specialize in transmitting signals exclusively for specific pathways (Figure 1i). Notch1 exemplifies this type of signaling: upon binding and activation by an extracellular ligand, Notch1 undergoes cleavage and liberation of the Intracellular Domain (ICN), which translocates to the nucleus and effects transcriptional activation [1]. However, most intracellular signal transduction is carried out by intermediates that are common to multiple biochemical pathways (Figure 1ii) [2]. For example, activation of ERK and p38 MAPK and associated pathways occurs in response to many different receptors [3,4]. For a cell functioning *in vivo* and *in situ*, a multitude of simultaneous cellular signals must be integrated into seamless, unified, non-contradictory responses. This generates the conceptual problem of how common intermediates can be utilized to communicate different specific signals, without mistaking information between different pathways that may or may not be concurrently active.

It has been hypothesized that the solution to this problem may largely reside in the protein- protein interaction (PPI) network [5], which is thought to incorporate all of the genetically encoded biochemistry in a cell [6]. In this network model of signal specificity, PPI constitute a biochemical ‘language’, in which proteins are members of an ‘alphabet’ that in joining together form ‘words’ instructing the cell to perform functions. Because many different PPI from different biochemical pathways occur simultaneously, cellular decisions are thought to rely on network principles that would integrate the qualitative and quantitative PPI-mediated signals, and translate them into specific responses.

## PPI Network Robustness and the Central-Lethality Rule

**Figure 2:**
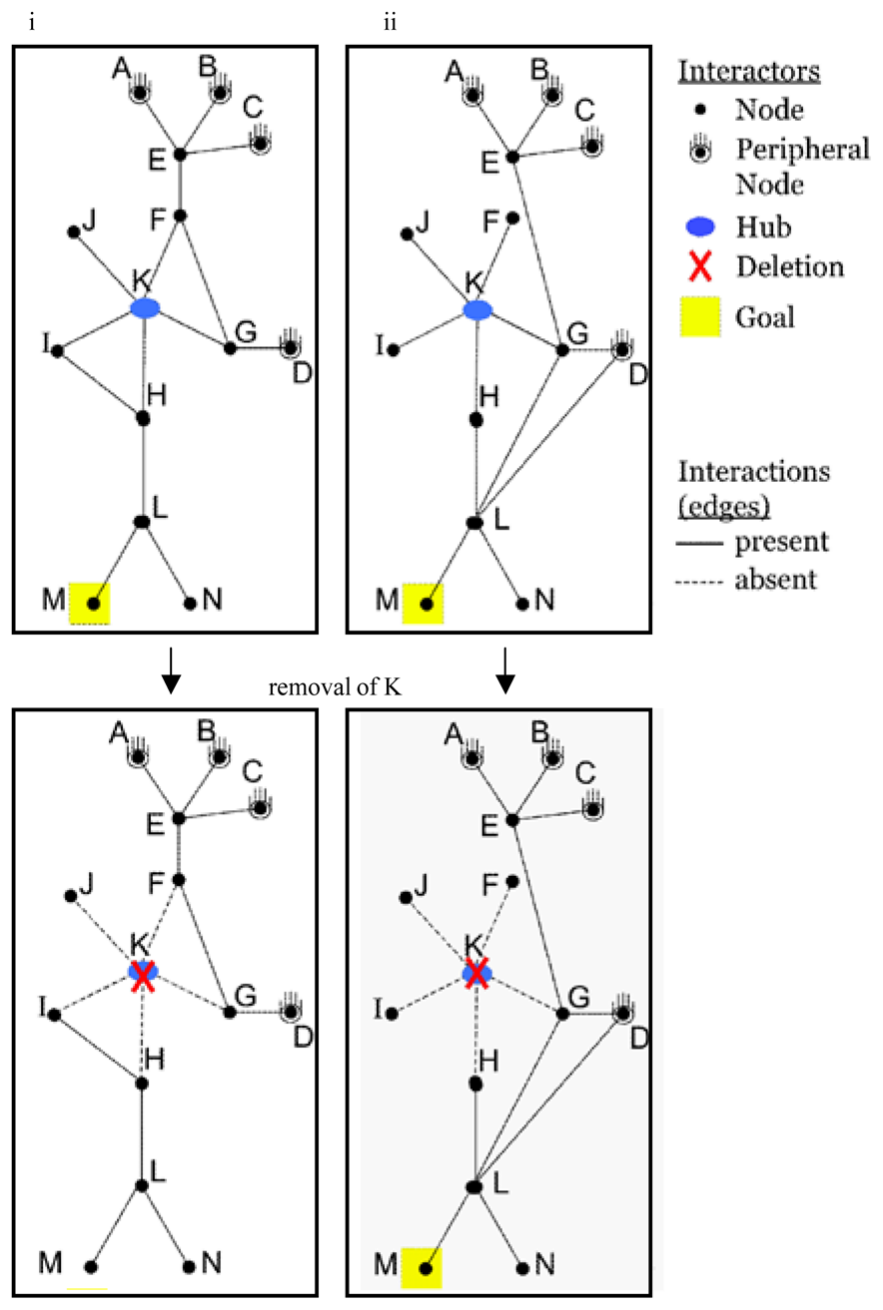
Two hypothetical physiologic PPI networks display distinct robustness upon deletion of a hub Two examples **(i–ii)** of node-edge diagrams for a hypothetical physiologic PPI network. (Top panels) Networks (i) and (ii) are identical except for 3 differences among the 15 interactions (edges) possessed by each. (Bottom panels) The consequence to each network upon genetic deletion of the gene encoding the protein for hub, K.

To understand how the PPI network might organize and direct many different signals, we must first consider the mechanisms by which it is predicted to transmit individual signals. A network can display emergent, synergistic properties that do not entirely account for by the individual contributions of its members. According to network theory, the PPI network is expected to display robustness, which describes a network’s stability in the face of changes to its members (individual proteins) or their functions [7,8]. Figure 2 displays two hypothetical PPI networks (Figure 2i and 2ii, top panels), each composed of 15 interactions (‘edges’) between 14 members (‘nodes’ A-N), including 4 receptors (initiators of signaling pathways, denoted as ‘peripheral nodes’ A-D), 1 protein with many interaction partners (‘hub’, K), and 1 goal partner (M), whose interaction induces a function. Except for three differences among the 15 interactions (edges), the two networks are otherwise identical. Hypothetically, if the gene for the hub protein, K, were deleted (Figure 2, bottom panels), this would eliminate the network’s ability to reach the goal (M) from any receptor in Figure 2i, but not Figure 2ii. Thus, the network in Figure 2ii is the most robust under this specific condition.

Experiments, mostly in the yeast *Saccharomyces cerevisiae*, have demonstrated that genetic deletion of protein hubs results in lethality more often than deletion of non-hub proteins, and this has become known as the central-lethality rule [9]. This may be partly explained in probabilistic terms. Because hubs participate in many more PPI than do average proteins, hubs might display an increased tendency to be involved in specific essential PPI that are required for cellular life [10]. However, it is thought that the network activity of hubs also contributes to their preferential essentiality. Hubs play a critical role in mediating communication within and between signaling pathways, as sub-centers of the many connections that together determine a network’s topology/connectivity. Therefore, deletion of a hub is thought to create a severe hole in the network that often cannot be bypassed by compensatory pathways [11,12]. The idea that the network activity of the genes encoding PPI hubs tends to be essential for cellular life is consistent with the hypothesis that there is a significant network component to PPI function.

## Redundant vs. Distributed Mechanisms of Network Robustness

Null mutation of any one of approximately 80% of yeast genes in *S. cerevisiae* was shown to bear no consequence for viability or growth rate in multiple different environments [13–15]. It is not thought that 80% of the yeast genome makes no contribution to the viability or growth rate; rather that the robustness of the genetic network allows compensatory mechanisms to sustain essential signals when one of the majorities of genes is lost. Subsequent studies showed that only a small fraction of this compensation were due to genetic redundancy, where a closely related family member performs the activity of the missing gene. Surprisingly, most of the compensation was due to distributed robustness, where unrelated genes diminish the phenotypic consequences of the network alteration [16,17]. Further work showed that a vast collection of chemical and environmental stress conditions could be identified in which nearly all genes were required for optimal growth in at least one condition, illustrating that the minimal genetic complement required for life was not equal to that which provided optimal/maximal function in a potentially variable environment [18]. Thus, in the yeast genome, genes display functional specificity, but the plasticity in the network allows some genes (or collections of genes) to compensate function when other network members are mutated [19,20].

Interestingly, in mice, the percent of knockout mutations in protein-encoding genes compatible with mouse viability has been estimated to be very close to that of yeast, ~80% [21]. Although not quantified, common experience dictates that many genetic knockouts display no phenotype, sometimes despite other experimental evidence that these genes perform specific functions [19,22]. Because of these and other examples and analyses, mutational robustness are considered a network property inherent to genomes [8]. In this regard, an outstanding issue of great interest emerges, whose resolution is severely limited by current technical and analytical capabilities: to what extent does genetic network robustness function directly through PPI network robustness, vs. through other genetic, epigenetic, and/or genomic mechanisms?

## Toward Physiologic PPI Network Analysis of Robustness and Signal Specificity

**Figure 3:**
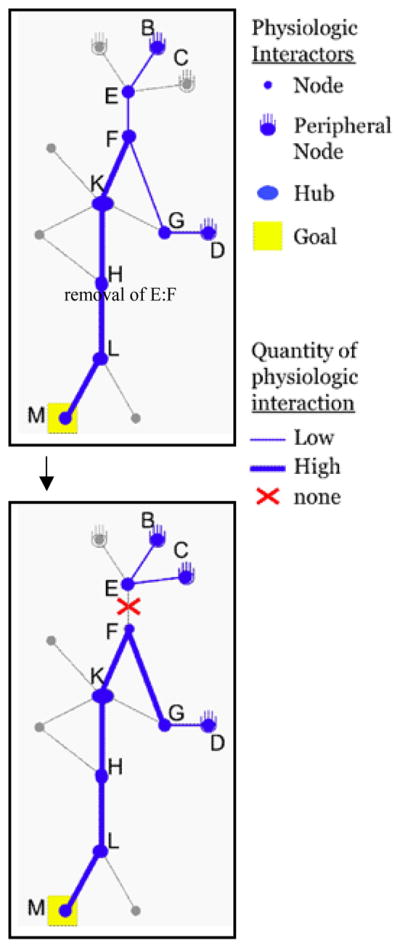
Robustness and signal specificity revealed when quantitative PPI information contributes to PPI network analysis (Top panel) Binary PPI data provides the network of all known possible interactions for this hypothetical PPI network, previously shown in Figure 2i (gray). Empirical, quantitative physiologic PPI information is overlaid (blue). (Bottom panel) One possible consequence to the network upon prevention or removal of the specific PPI, E:F.

The current battery of protein interaction methodologies is designed to report and archive binary PPI data, wherein the presence or absence of interaction between two specific proteins is noted [23]. Well-known strategies with which to approach PPI experiments include Glutathione S-Transferase (GST)-fusion protein pull-down (PD) [24], co-Immunoprecipitation (IP) with subsequent Western blotting [25] or mass spectrometry-based identification of binding partners [26,27], Blue Native-Polyacrylamide Gel Electrophoresis (BN-PAGE) [28], Fluorescence Resonance Energy Transfer (FRET) [29], and yeast two-hybrid [30]. The binary data thus generated can be retrieved from databases and assembled into PPI network maps, large and smallest of various scales, but similar in concept to Figure 2. However, PPI network analysis lags behind genetic network analysis platforms because of the lack of quantitative data obtainable under physiologic conditions. Therefore, current state-of-the-art PPI maps constructed from archived data display possible PPI, in the same way a highway map shows roads, but not traffic/activity. Significant advances are needed to generate the technological and analytical ability to quantify large collections of PPI and reveal the extent to which they actually occur under specific physiological conditions in primary cells [31]. Figure 3 illustrates what such data might look like on a small scale, if the technology existed to generate it, for the same hypothetical PPI network shown previously. The possible PPI representing current data capabilities is shown in grey, overlaid with physiologic quantification data in blue that indicates the PPI level under a specific condition. First, physiologic conditions can control which proteins (nodes) are present due to differential gene expression and other post-transcriptional mechanisms of protein abundance. In Figure 3, this is evident because of all the proteins that could potentially participate in PPI (grey with no blue overlay), only a subset is present under this hypothetical physiological condition (blue overlay). Second, PPI is subject to a plethora of regulatory processes *in vivo*: competition between multiple ligands, sequestration, compartmentalization, glycosylation, allosterism, binding site alteration (phosphorylation, de-phosphorylation, ubiquitination), and others. Therefore, it is easily imagined that in one physiological condition a PPI might be engaged (Figure 3, top panel), while under different conditions that PPI may not occur (Figure 3, bottom panel, showing lack of the PPI, E:F).

Here, it will be illustrated how measuring the changes in the quantity of an interaction between protein pairs could reveal physiologic PPI signatures of network robustness and signal specificity. Under one hypothetical physiologic condition (Figure 3, top), both B and D pathways lead to recruitment of F, and therefore the quantity of signal from F to the goal M is high. However under the second hypothetical physiologic condition (Figure 3, bottom) where BCE forms a constitutive complex that inhibits formation of E:F, only receptor D continues to signal to M. The signal from F to M might be reduced due to the lack of input from the B pathway (not shown); but alternatively, loss of E:F might liberate more F to interact between G and K, enhancing the total signal strength through D to compensate for the lack of signaling through B. Thus, summarizing both Figures 2 and 3, the receptors A-D share signaling specificity for M through a PPI network involving shared intermediates. Network robustness depends on the distribution of connectivity [32] (Figure 2), and the plasticity of signaling strength determined through multiple pathways (Figure 3). The degree to which the proteins (nodes) involved originate from related vs. unrelated genes would determine the role of redundant vs. distributed mechanisms of robustness.

## Extending the Reach of Technology to Profile Physiologic PPI Networks in Health and Disease

The summation of all possible PPI and other molecular interactions is collectively termed the ‘interactome’ [33]. As a field of study, Interactomics represents a frontier in which progress is currently limited by both assay and analytical tools, to a degree beyond that which applies to Genomics or Proteomics [34]. Whereas these latter fields focus mostly on the identity and expression level of molecular species, the output information of these sciences is the input information for Interactomics. A complete Interactomic profile, which does not yet exist, would measure all possible combinations of interactions between molecules as reported by other ‘omics’ methods, and add exponential matrix-level interaction complexity that would be considerably more data-intensive than either Genomics or Proteomics parent sciences. In practice, such a level of interaction profiling is not realistically imminent. However, to progress in this direction, there is great interest in the generation of assay and analytical tools that improve the accessibility of molecular interactions to experimentation, diagnosis, pharmacology, and medicine [33,34].

A central rationale for the pursuit of improved Interactomic technologies is that PPI engagement/activity is likely distinct in healthy vs. diseased states, since PPI relay the signals that may be associated with these opposite outcomes. To better understand these signals, the field needs technologies with better capabilities to observe large swaths of PPI networks, ideally from samples as small as those routinely obtained in the clinic. Two of the most promising technological avenues in this regard involve Mass Spectrometry (MS) and microsphere-based PPI analysis. Recent studies using MS produced the first quantitative estimate of a proteomic signature for a human cell line, by coupling empirical protein quantification strategies with computational and statistical modeling [35]. Applying this level of protein quantification to protein interaction quantities will not be trivial, and will represent a significant advance toward physiologic network PPI analysis. Meanwhile, other recent experiments have begun to approach PPI measurement using high-sensitivity ELISA-style methodology via singleplex or multiplex microspheres [36–40]. The strength of this latter approach lies in its compatibility with small-volume samples from primary tissue sources including patient samples, as well as high-throughput multi-well formatting and potential applicability to drug screening. Further development of both MS and multiplex microsphere-based strategies is likely to prove the unique strengths of both approaches to be complementary. If MS could be used to identify the most comprehensive Interactomic PPI set possible, then that could inform the composition of smaller-scale multiplex microsphere-based micro-assays whose current maximum is limited to ~500 primary analytes. Those MS-informed high-sensitivity multiplex PPI assays could be used to target specific signaling pathways, to visualize relevant subsets of PPI networks in patient samples, or to design drug screening strategies that evaluate effects on large collections of PPI instead of single protein activities.

## Concluding Remarks

Considering the PPI that mediates signal transduction from a network biology perspective provides a model for how cellular biochemistry may control signal robustness and specificity. Progress in understanding gene networks supplies a template for the concepts hypothesized to emerge from physiologic PPI networks, including potentially significant roles in signal transduction for redundant vs. distributed mechanisms of robustness. The achievement of new advances in technology to enable physiologic network PPI analysis may not only lead to increased understanding of signalling mechanisms, but also provides network PPI profile for clinical medicine, and new experimental platforms for drug discovery and pharmacology.

## References

[1] Penton AL, Leonard LD, Spinner NB (2012). Notch signaling in human development and disease. Semin Cell Dev Biol.

[2] Pawson T (2004). Specificity in signal transduction: from phosphotyrosine-SH2 domain interactions to complex cellular systems. Cell.

[3] Werlen G, Hausmann B, Naeher D, Palmer E (2003). Signaling life and death in the thymus:timing is everything. Science.

[4] Ashwell JD (2006). The many paths to p38 mitogen-activated protein kinase activation in the immune system. Nat Rev Immunol.

[5] Hunter T (2000). Signaling--2000 and beyond. Cell.

[6] Komarova NL, Zou X, Nie Q, Bardwell L (2005). A theoretical framework for specificity in cell signaling. Mol Syst Biol.

[7] Chen BS, Wang YC, Wu WS, Li WH (2005). A new measure of the robustness of biochemical networks. Bioinformatics.

[8] Gardner A, Kalinka AT (2006). Evolution of mutational robustness in the yeast genome: a link to essential genes and meiotic recombination hotspots. J Theor Biol.

[9] Jeong H, Mason SP, Barabasi AL, Oltvai ZN (2001). Lethality and centrality in protein networks. Nature.

[10] He X, Zhang J (2006). Why do hubs tend to be essential in protein networks?. PLoS Genet.

[11] Pang K, Sheng H, Ma X (2010). Understanding gene essentiality by finely characterizing hubs in the yeast protein interaction network. Biochem Biophys Res Commun.

[12] Ning K, Ng HK, Srihari S, Leong HW, Nesvizhskii AI (2010). Examination of the relationship between essential genes in PPI network and hub proteins in reverse nearest neighbor topology. BMC Bioinformatics.

[13] Smith V, Chou KN, Lashkari D, Botstein D, Brown PO (1996). Functional analysis of the genes of yeast chromosome V by genetic footprinting. Science.

[14] Winzeler EA, Shoemaker DD, Astromoff A, Liang H, Anderson K (1999). Functional characterization of the S. cerevisiae genome by gene deletion and parallel analysis. Science.

[15] Giaever G, Chu AM, Ni L, Connelly C, Riles L (2002). Functional profiling of the Saccharomyces cerevisiae genome. Nature.

[16] Wagner A (2000). Robustness against mutations in genetic networks of yeast. Nat Genet.

[17] Kafri R, Levy M, Pilpel Y (2006). The regulatory utilization of genetic redundancy through responsive backup circuits. Proc Natl Acad Sci USA.

[18] Hillenmeyer ME, Fung E, Wildenhain J, Pierce SE, Hoon S (2008). The chemical genomic portrait of yeast: uncovering a phenotype for all genes. Science.

[19] Wolfe K (2000). Robustness--it’s not where you think it is. Nat Genet.

[20] Hartman JL, Garvik B, Hartwell L (2001). Principles for the buffering of genetic variation. Science.

[21] Wilson L, Ching YH, Farias M, Hartford SA, Howell G (2005). Random mutagenesis of proximal mouse chromosome 5 uncovers predominantly embryonic lethal mutations. Genome Res.

[22] Saga Y, Yagi T, Ikawa Y, Sakakura T, Aizawa S (1992). Mice develop normally without tenascin. Genes Dev.

[23] Kuroda K, Kato M, Mima J, Ueda M (2006). Systems for the detection and analysis of protein-protein interactions. Appl Microbiol Biotechnol.

[24] Smith DB, Johnson KS (1988). Single-step purification of polypeptides expressed in Escherichia coli as fusions with glutathione S-transferase. Gene.

[25] Phizicky EM, Fields S (1995). Protein-protein interactions: methods for detection and analysis. Microbiol Rev.

[26] Conrotto P, Yakymovych I, Yakymovych M, Souchelnytskyi S (2007). Interactome of transforming growth factor-beta type I receptor (TbetaRI): inhibition of TGFbeta signaling by Epac1. J Proteome Res.

[27] Tu LC, Yan X, Hood L, Lin B (2007). Proteomics analysis of the interactome of N-myc downstream regulated gene 1 and its interactions with the androgen response program in prostate cancer cells. Mol Cell Proteomics.

[28] Swamy M, Siegers GM, Minguet S, Wollscheid B, Schamel WW (2006). Blue native polyacrylamide gel electrophoresis (BN-PAGE) for the identification and analysis of multiprotein complexes. Sci STKE.

[29] Jares-Erijman EA, Jovin TM (2006). Imaging molecular interactions in living cells by FRET microscopy. Curr Opin Chem Biol.

[30] Parrish JR, Gulyas KD, Finley RL (2006). Yeast two-hybrid contributions to interactome mapping. Curr Opin Biotechnol.

[31] Mueller M, Martens L, Apweiler R (2007). Annotating the human proteome: beyond establishing a parts list. Biochim Biophys Acta.

[32] Balaji S, Iyer LM, Aravind L, Babu MM (2006). Uncovering a hidden distributed architecture behind scale-free transcriptional regulatory networks. J Mol Biol.

[33] Rual JF, Venkatesan K, Hao T, Hirozane-Kishikawa T, Dricot A (2005). Towards a proteome-scale map of the human protein-protein interaction network. Nature.

[34] Morell M, Aviles FX, Ventura S (2009). Detecting and interfering protein interactions: towards the control of biochemical pathways. Curr Med Chem.

[35] Beck M, Schmidt A, Malmstroem J, Claassen M, Ori A (2011). The quantitative proteome of a human cell line. Mol Syst Biol.

[36] Schrum AG, Gil D, Dopfer EP, Wiest DL, Turka LA (2007). High-sensitivity detection and quantitative analysis of native protein-protein interactions and multiprotein complexes by flow cytometry. Sci STKE.

[37] Schrum AG (2009). Visualization of multiprotein complexes by flow cytometry. Curr Protoc Immunol.

[38] Davis TR, Schrum AG (2010). IP-FCM: Immunoprecipitation Detected by Flow Cytometry. J Vis Exp.

[39] Smith SEP, Bida AT, Davis TR, Sicotte H, Patterson SE (2012). IP-FCM measures physiologic protein-protein interactions modulated by signal transduction and small-molecule drug inhibition. PLoS One.

[40] Bida AT, Gil D, Schrum AG (2012). Multiplex IP-FCM (immunoprecipitation-flow cytometry): Principles and guidelines for assessing physiologic protein-protein interactions in multiprotein complexes. Methods.

